# A high-quality apple flower detection dataset for precision agriculture

**DOI:** 10.3389/fpls.2026.1927541

**Published:** 2026-08-26

**Authors:** Dandan Wang, Bo Wang

**Affiliations:** 1 College of Communication and Information Engineering, Xi’an University of Science and Technology, Xi’an, Shaanxi, China; 2 Xi’an Key Laboratory of Network Convergence Communication, Xi’an, Shaanxi, China; 3 School of Automation Science and Engineering, Faculty of Electronic and Information Engineering, Xi’an Jiaotong University, Xi’an, Shaanxi, China

**Keywords:** agricultural object detection, apple flower, image dataset, orchard, precision agriculture

## Abstract

Accurate detection of apple flowers and buds at the ground level is a prerequisite for precision orchard management, yet it remains constrained by the scarcity of densely annotated, in-canopy datasets. In this study, we present a dataset for apple flower and bud detection collected in a commercial apple orchard in Shaanxi Province, China. The dataset comprises 3,117 images and 52,181 annotated instances, capturing the structural complexity of tree canopies under variable illumination and partial occlusion. A key characteristic of this dataset is the concurrent presence of fully opened flowers and unopened buds, alongside considerable morphological variability arising from differential petal loss. High-precision bounding boxes distinguish the two classes, with annotations provided in XML, YOLO (TXT), and COCO (JSON) formats to ensure broad compatibility. Standardized train/validation/test splits (8:1:1) are supplied, and baseline performance is established using representative architectures, including YOLOv11n, RT-DETR, and DEIMv2. Evaluation results reveal a clear performance gap between buds and flowers, underscoring the inherent detection difficulty of buds due to morphological ambiguity and occlusion. By providing this high-quality dataset, we aim to support the development of robust vision algorithms for early bloom monitoring, facilitating critical management decisions such as site-specific flower thinning and yield estimation.

## 1 Introduction

Accurate detection of apple flowers and flower buds in orchards is a prerequisite for precision agriculture applications such as bloom intensity estimation, early yield prediction, and site-specific flower thinning (Dias et al., 2018). Conventional manual assessments are labor-intensive, subjective, and poorly scalable across large orchards, creating strong demand for automated, vision-based monitoring solutions (Tian et al., 2020).

Deep learning-based object detectors, including the YOLO series and Faster R-CNN, have achieved promising results in agricultural detection tasks (Lu and Young, 2020; Häni et al., 2020). However, their practical deployment in orchards depends heavily on annotated datasets that reflect the structural complexity and environmental variability of real-world production systems. Publicly available datasets targeting the apple flowering or flower-bud stage remain scarce; one notable example is AriAplBud, a multi-growth-stage apple flower bud dataset acquired from an aerial perspective (Yuan, 2024). Although valuable for large-scale phenotyping, such aerial datasets differ substantially from the close-range, in-canopy imaging conditions encountered by ground-based detection platforms, such as handheld devices, ground robots, or robotic sprayers.

Ground-level flower and bud detection presents distinct challenges, including small target size, dense floral clustering, partial occlusion by foliage, variable illumination within the tree canopy, and considerable morphological variability arising from differential petal loss in open flowers (Bhattarai et al., 2020; Farjon et al., 2020; Estrada et al., 2024; Zhou et al., 2024; Mu et al., 2023; Li et al., 2022). Moreover, many existing studies focus exclusively on fully opened flowers, neglecting unopened buds despite their importance for early bloom monitoring and management decision-making (Sun et al., 2021). Although flower and bud instances may coexist in certain images of multi-stage datasets, publicly available datasets that specifically target ground-level, in-canopy detection with explicit class separation between open flowers and unopened buds remain limited.

To complement existing benchmarks, we present an apple flower and bud detection dataset collected in a commercial apple orchard in Shaanxi Province, China, on March 28, 2021, a representative date within the early flowering stage following first bloom in this region, when both fully opened flowers and unopened buds were commonly present in the canopy. The dataset comprises 3,117 images and 52,181 annotated instances, with high-precision bounding boxes distinguishing flowers from buds. Annotations are provided in XML, YOLO (TXT), and COCO (JSON) formats, accompanied by standardized train/validation/test splits and baseline results established using representative detection architectures (e.g., YOLOv11n, RT-DETR). By releasing this dataset, we aim to provide a robust, ground-level benchmark that bridges the gap between aerial phenotyping and in-canopy monitoring, thereby supporting the development of reliable vision systems for precision orchard management.

## 2 Value of the data

### 2.1 Diverse orchard scenarios for real-world robustness

The dataset was collected in a commercial apple orchard under uncontrolled natural conditions, encompassing variations in illumination, viewing angles, occlusion by foliage, and floral clustering. This *in-situ* acquisition strategy reflects the variability encountered in real-world orchard deployments, improving the generalization capability of trained detection models.

### 2.2 High-quality annotations for object detection tasks

All images are manually annotated with high-quality bounding boxes distinguishing fully opened apple flowers from unopened buds. The two-class scheme complements existing agricultural datasets that primarily emphasize fruit or aerial phenotyping, and supports fine-grained blossom density estimation, early flowering monitoring, and yield prediction in precision orchard management.

### 2.3 Framework-ready annotation suite

To facilitate seamless integration with the computer vision community, the dataset is released with annotations in three dominant formats: XML, YOLO (TXT), and COCO (JSON). This multi-format provision eliminates the need for tedious format conversions, enabling direct deployment on mainstream architectures (e.g., YOLO-series, Faster R-CNN, RT-DETR). Standardized train/validation/test splits are included to promote experimental reproducibility and ensure fair cross-study comparisons.

### 2.4 Publicly available resource for agricultural AI research

By openly releasing the complete dataset along with annotations and baseline detection results, this work provides a valuable benchmark for the agricultural computer vision community. As a ground-level, in-canopy flower and bud detection benchmark, it facilitates future research on orchard automation, phenotyping, and smart farming systems.

## 3 Materials and methods

**Figure 1:**
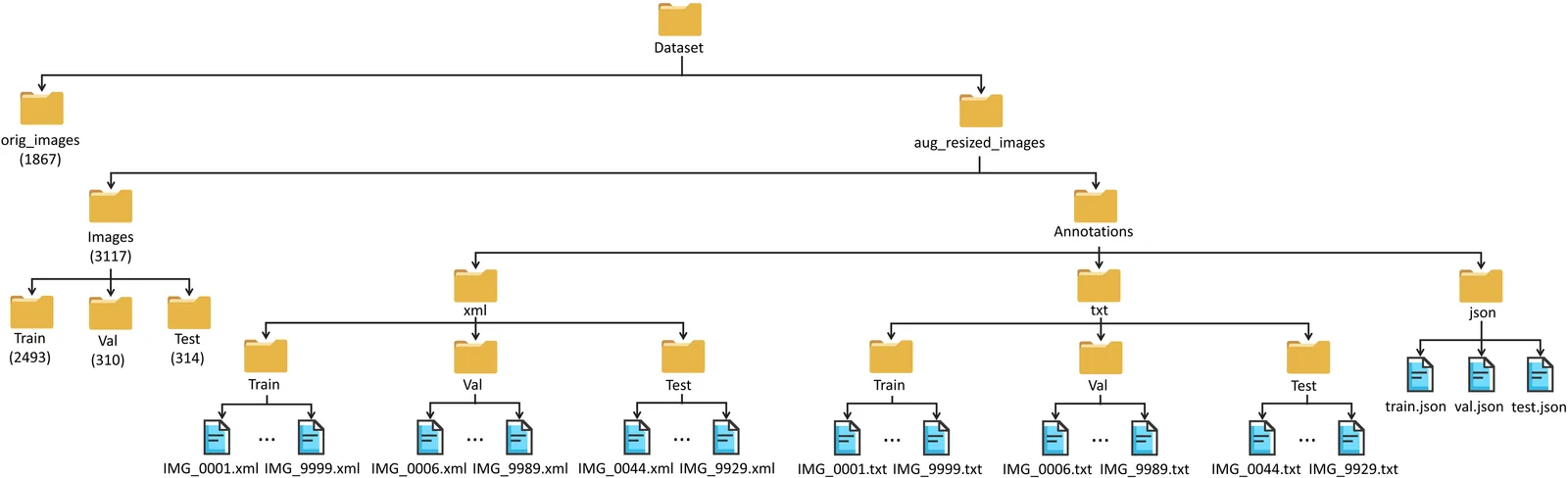
Hierarchical organization of dataset.

This study introduces a high-density dataset supporting ground-level detection of apple flowers and buds in unstructured orchard environments, with its repository architecture detailed in **Figure 1**. The dataset root comprises two main branches, orig_images (raw high resolution images) and aug_resized_images (augmented resized images and annotations). Each branch maintains consistent train/val/test splits across all annotation formats. This multi-format, ready-to-use configuration eliminates the need for tedious format conversions, allowing researchers to directly benchmark architectures ranging from YOLO-series models to Transformer-based detectors.

### 3.1 Data collection

**Figure 2:**
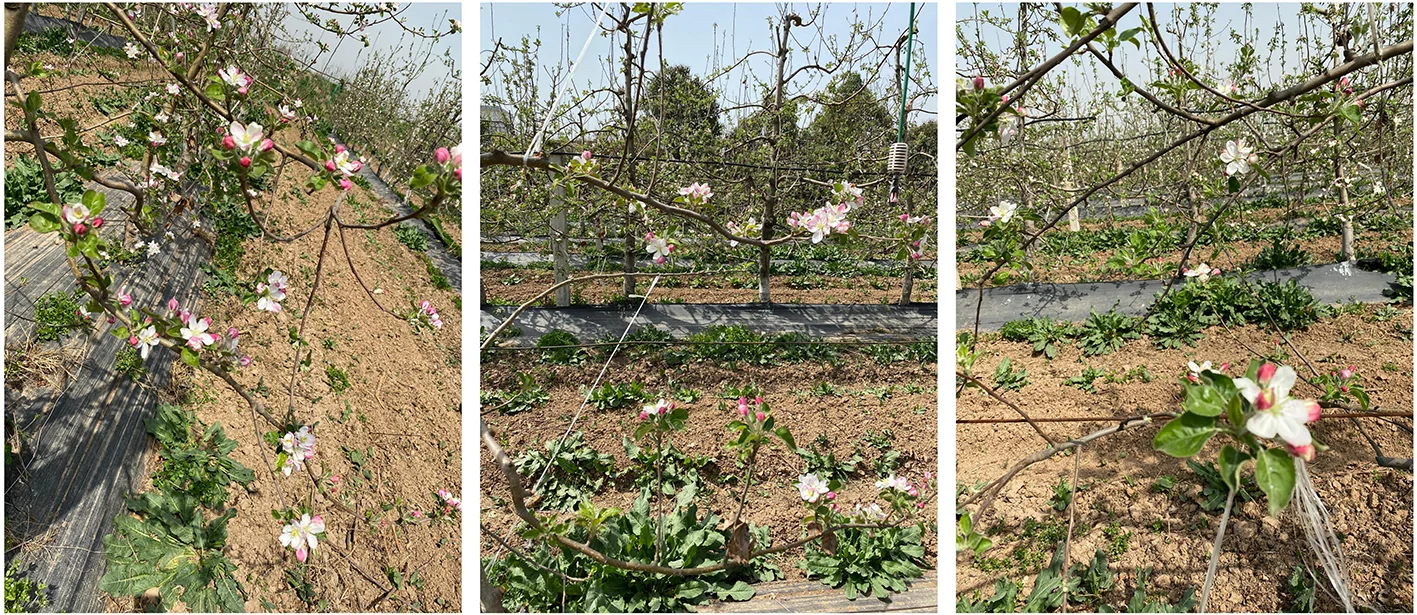
Orchard scenes.

**Figure 3:**
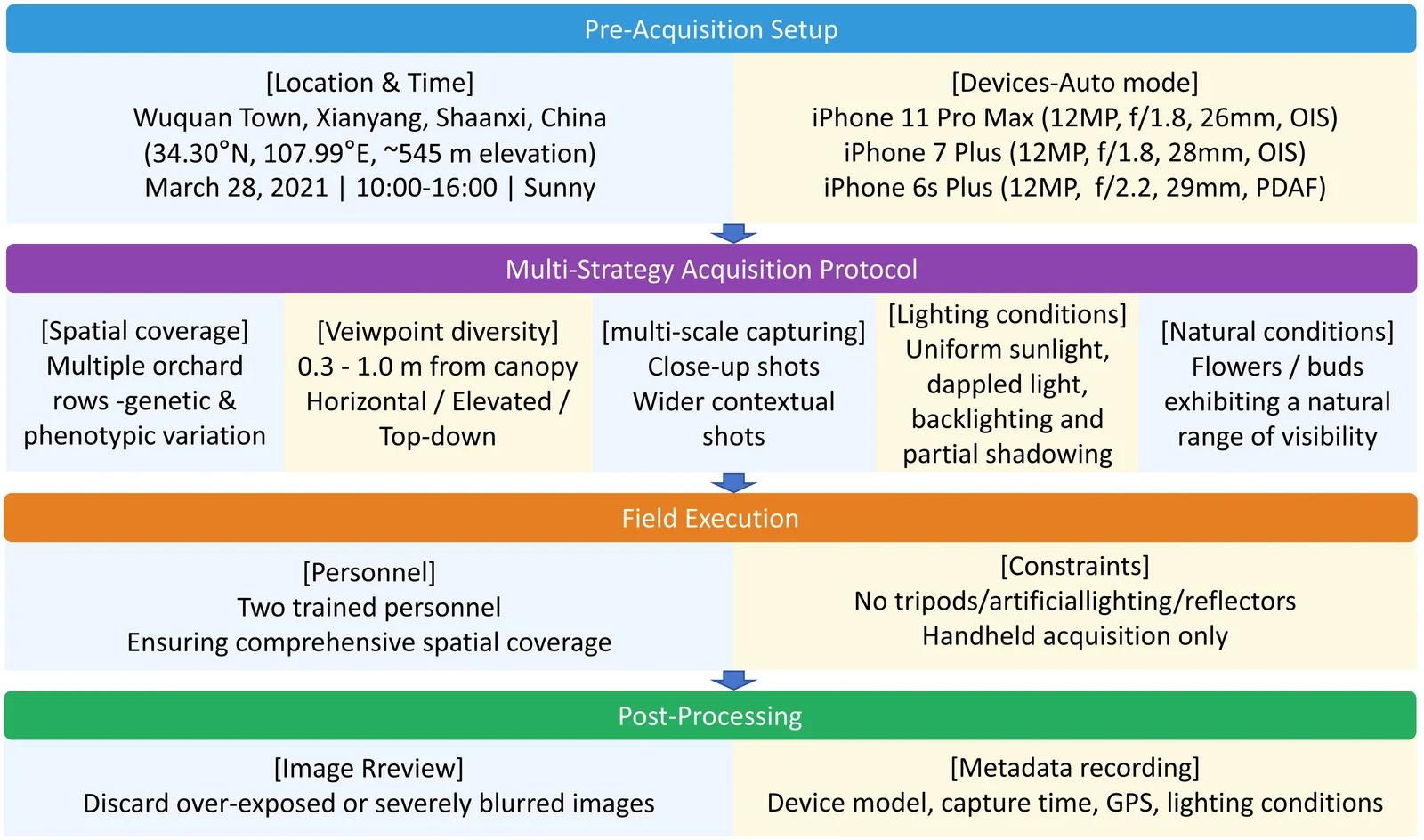
Image acquisition workflow.

All images were collected in a commercial apple orchard located in Wuquan Town, Xianyang City, Shaanxi Province, China (34.30°N, 107.99°E; elevation: approximately 545 m). The orchard scenes are shown in **Figure 2**. The orchard cultivar was ‘Granny Smith’ (*Malus domestica* Borkh.), and imaging was conducted on March 28, 2021—a representative date within the early full-bloom stage following first bloom in this region—under sunny weather conditions between 10:00 a.m. and 4:00 p.m. (local time). Three consumer-grade smartphone devices were used to simulate practical ground-level acquisition conditions, reflecting the diversity of cameras typically available for orchard management: an iPhone 11 Pro Max, equipped with a 12-megapixel wide CMOS sensor (f/1.8 aperture, 26.0 mm focal length, dual pixel autofocus, optical image stabilization), an iPhone 7 Plus, equipped with a 12-megapixel CMOS sensor (f/1.8 aperture, 28.0 mm focal length, hybrid autofocus, optical image stabilization), and an iPhone 6s Plus, equipped with a 12-megapixel CMOS sensor (f/2.2 aperture, 29.0 mm focal length, phase detection autofocus). All devices were set to automatic mode to reflect real-world deployment scenarios. The original image resolution was 3024 × 4032 pixels for all devices. These consumer-grade smartphones were selected to introduce variability in sensor and image processing characteristics while remaining representative of readily available, non-specialized imaging devices used in field-based agricultural research. Image acquisition details are provided in **Table 1**. The complete image acquisition workflow, encompassing pre-acquisition setup, multi-strategy acquisition protocol, field execution, and post-processing, is illustrated in **Figure 3**.

**Table 1:** Image capture details.

Parameter	Specification
Manufacture	Apple
Equipment	iPhone 11 Pro Max/iPhone 7 Plus/iPhone 6s Plus
Flash mode	Off
Image dimensions	3024×4032 pixels
Color depth	24-bit (RGB)
Image count	716 (iPhone 11 Pro Max) 486 (iPhone 7 Plus) 665 (iPhone 6s Plus)
Total images	1867

To ensure the dataset reflects the complexity of unstructured orchard environments, images were acquired following a protocol designed to maximize variability in viewing perspectives, lighting, and occlusion:

1. Imaging was conducted across multiple rows within the orchard to capture genetic and phenotypic variations inherent in the ‘Granny Smith’ cultivar.
2. The camera was positioned to simulate the near-range perspectives encountered by ground-based automated systems (e.g., automated thinning robots or low-mounted sprayers), while respecting the physical constraints of handheld data acquisition. Images were captured at distances ranging from 0.3 to 1.0 meters from the target canopy. A combination of horizontal, elevated, and top-down viewing angles was employed to replicate the diverse orientations encountered during mechanical operations.
3. To account for the spatial distribution of flowers and buds, both close-up shots (focusing on individual or small clusters of targets) and wider contextual shots capturing local canopy sections or entire fruiting branches were acquired. This multi-scale approach ensures the dataset is robust to targets of varying apparent sizes within practical detection ranges.
4. Rather than restricting specific lighting conditions, images were collected under the full spectrum of natural illumination present between 10:00 and 16:00. This intentionally included uniform sunlight, high-contrast scenarios with dappled light filtering through the canopy, and challenging conditions such as strong backlighting and partial shadowing caused by leaves or branches.
5. Images were captured under natural orchard conditions without artificial manipulation of branch or leaf positions. Consequently, the dataset inherently contains flowers and buds exhibiting a natural range of visibility, from fully exposed targets to those partially obscured by foliage, woody branches, or adjacent blossoms.

**Figure 4:**
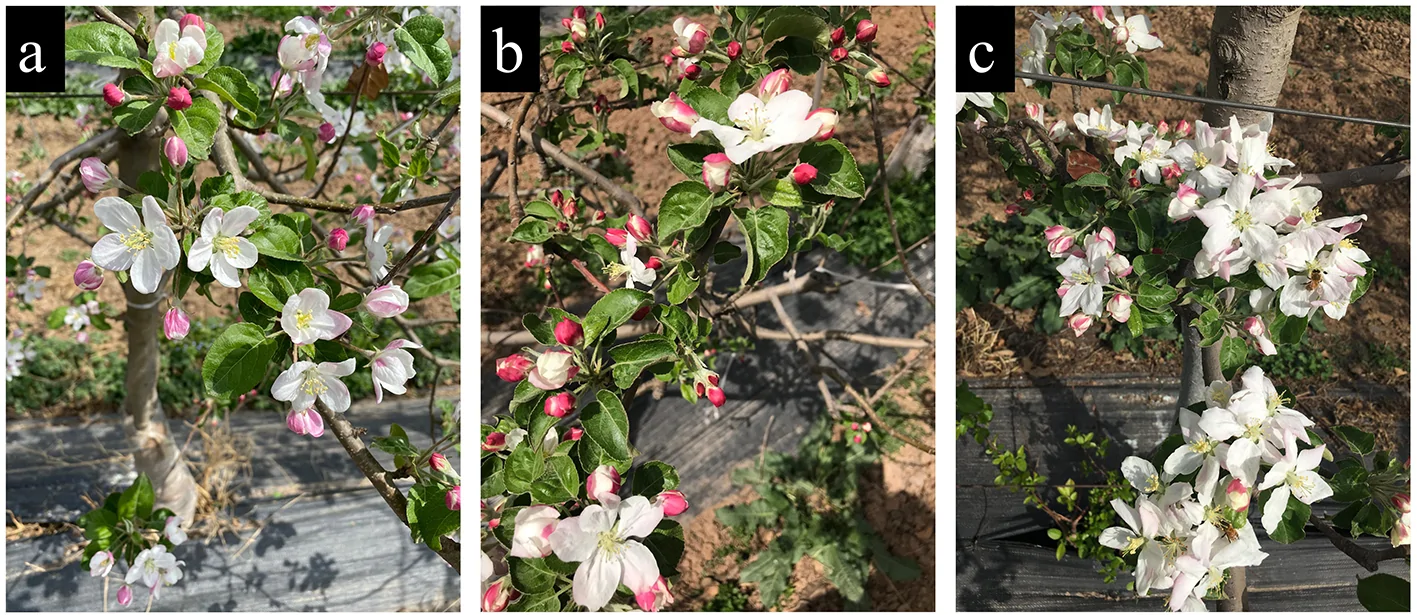
Images captured by different devices. **(A)** Images captured by iPhone 11 Pro Max; **(B)** Images captured by iPhone 7 Plus; **(C)** Images captured by iPhone 6s Plus.

Two trained personnel conducted the acquisition to ensure comprehensive spatial coverage. No artificial lighting, reflectors, or tripods were used, ensuring the dataset accurately represents the visual conditions encountered during routine orchard monitoring. Examples of images captured by different devices are illustrated in **Figure 4**.

Following the acquisition protocol, images were stored in JPEG format. An initial set of more than 2,800 images was captured during the data collection. To ensure data quality, images exhibiting severe motion blur, critical focus errors, or extreme overexposure were manually discarded. After this quality control process, a set of 1,867 high-quality images remained for annotation and subsequent benchmarking.

### 3.2 Data annotation

**Figure 5:**
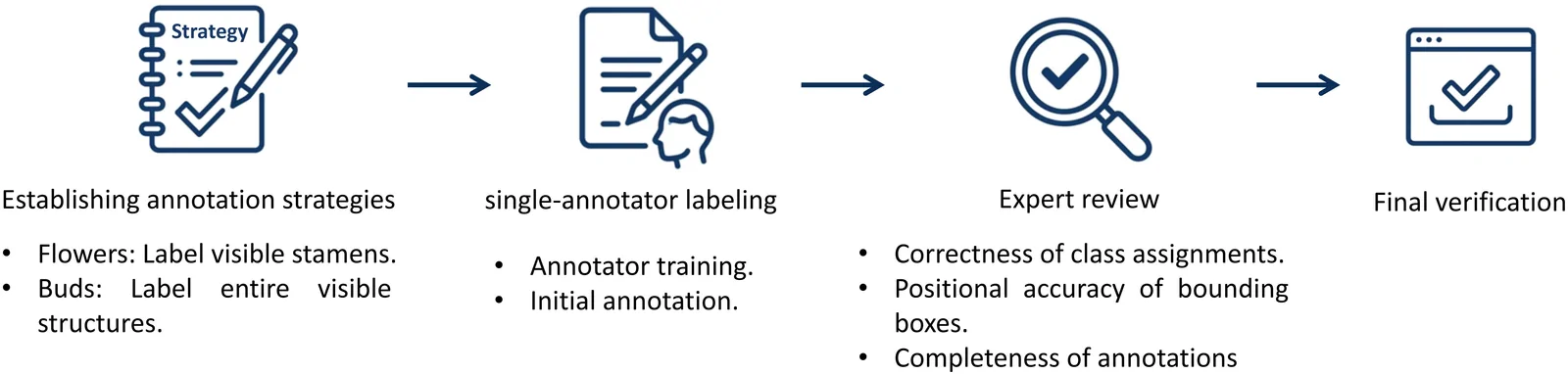
Data annotation workflow.

All images were uniformly resized to 605 × 807 pixels (maintaining the original aspect ratio) prior to annotation to reduce computational overhead and standardize input dimensions. Annotation was performed using the open-source graphical image annotation tool LabelImg​ (https://github.com/tzutalin/labelImg), with bounding boxes saved in XML format. The annotation workflow is shown in **Figure 5**.

#### 3.2.1 Annotation strategy

A two-class annotation scheme was adopted: flower and bud.

Flower annotation: Unlike conventional approaches that annotate the entire corolla, flowers were annotated by focusing on the central stamen. This strategy was deliberately chosen to address two practical challenges in orchard imagery: (1) severe occlusion and overlap among petals in densely clustered blossoms, where drawing bounding boxes around entire flowers inevitably leads to excessive box overlap and ambiguous labels; and (2) morphological variability caused by natural petal wilting or partial detachment. By anchoring the annotation to the stamen, the dataset maintains a consistent representation of flower presence even when petals are absent or fragmented.

Bud annotation: Unopened flower buds were annotated by drawing bounding boxes around the entire visible structure, encompassing both the bracts and the developing floral primordia.

Scale-aware annotation constraint: To address the challenge of distant targets within the deep canopy, a minimum resolvable feature criterion was enforced. Instances appearing as indistinct specks lacking discernible botanical structures (e.g., stamen) were excluded from annotation, regardless of their presumed class. Annotation was restricted to targets where the defining morphological characteristics—visible stamens for flowers or tightly closed petals for buds—remained taxonomically recognizable despite scale reduction. This protocol mitigates the inclusion of ambiguous specks that could degrade model training and ensures that the dataset reflects a realistic upper bound of taxonomic recognition.

Risk mitigation and annotation integrity: Despite the structured protocol, large-scale annotation introduces risks related to human subjectivity and perceptual ambiguity, particularly for edge cases such as partially occluded flowers or buds in transitional developmental stages. To mitigate these risks, strict botanical definitions were enforced: a flower was labeled only if stamens were distinctly visible, whereas buds were characterized by tightly closed petals. Ambiguous instances that could not be confidently classified were excluded from the dataset. To further reduce noise from perceptual uncertainty, the protocol formally defined ignore regions for targets that were severely truncated, blurred, or occluded beyond taxonomic recognition; these regions were excluded from supervision and not treated as false negatives during evaluation. These measures, reinforced by the hierarchical verification pipeline described below, ensure the reliability of all annotations.

**Figure 6:**
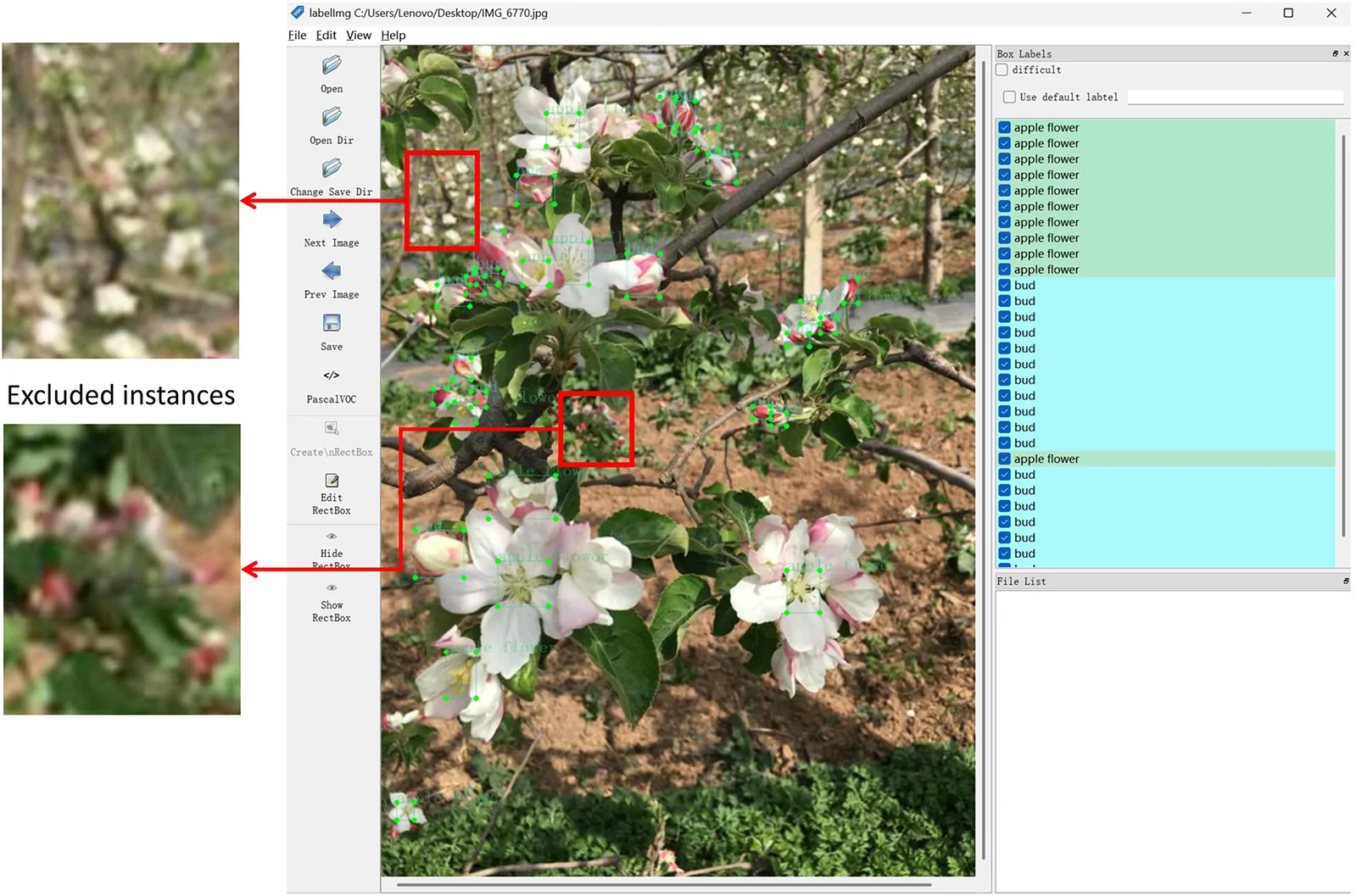
Illustration of the scale-aware annotation protocol. Valid annotations: bounding boxes are applied only when defining morphological characteristics (visible stamens for flowers or tightly closed petals for buds) are taxonomically recognizable. Excluded instances: targets are intentionally omitted if they appear as indistinct specks, are severely truncated, or are blurred beyond taxonomic recognition, thereby defining the ignore regions of the dataset.

An example of annotation, illustrating both valid bounding boxes and intentionally excluded instances (due to insufficient size or blur), is provided in **Figure 6**.

#### 3.2.2 Quality control protocol

To ensure annotation consistency, accuracy, and biological plausibility, a rigorous three-stage quality control protocol was implemented:

Stage 1: Standardized single-annotator protocol.

A single trained annotator performed all initial labeling to eliminate inter-observer variability. Prior to formal annotation, the annotator underwent a structured training process consisting of: (a) a detailed review of annotation guidelines defining the bounding box methodology (stamen-centric for flowers, whole-structure for buds); (b) three iterative rounds of practice labeling on a held-out subset of 100 images, with discrepancies discussed and resolved collaboratively with the research team; and (c) the establishment of explicit decision rules for edge cases:

1. Size and clarity thresholds: Targets were excluded if bounding box areas fell below an empirically derived lower bound of approximately 20 pixels—a scale at which stamen filaments or bud scales become optically irresolvable at the annotated resolution of 605 × 807 pixels. Equally, instances exhibiting severe motion or defocus blur that obscured these defining morphological features were discarded. This thresholding criterion was instituted to prevent the introduction of low-confidence, noisy labels arising from perceptually ambiguous specks, thereby safeguarding detector training against taxonomically unreliable supervision signals.
2. Occlusion handling: Targets were annotated if the stamen or bud body was partially visible; those with less than approximately 20% visibility were discarded to avoid ambiguity.
3. Overlapping instances: The tightest possible bounding box was drawn around each discernible stamen or bud, prioritizing the separation of adjacent targets even in dense clusters.

Stage 2: Expert review by pomologist.

Upon completion of initial annotation, all 1,867 images were systematically reviewed by an agricultural expert specializing in pomology. The expert independently verified: (a) the correctness of class assignments (flower vs. bud); (b) the positional accuracy of bounding boxes relative to the defined anatomical landmarks; and (c) the completeness of annotations (i.e., no missed targets). The expert also validated the stamen-centric annotation strategy, confirming its biological validity for representing flower presence irrespective of petal status. Annotations identified as erroneous, ambiguous, or incomplete were either corrected by the expert or excluded from the dataset.

Stage 3: Final verification.

After expert corrections, a final verification pass was conducted by the original annotator to ensure that all expert-requested modifications were properly implemented. This iterative refinement process resulted in a highly curated dataset of 1,867 images, optimized for training robust object detection models in complex orchard environments.

#### 3.2.3 Preliminary data statistics

The dataset comprises 1,867 images and 39,751 annotated instances, including 26,536 buds and 13,215 apple flowers. A notable class imbalance exists, with buds outnumbering flowers by approximately 2:1. To mitigate this imbalance, a targeted data augmentation strategy was employed prior to model training, as described in Section 3.3.

### 3.3 Dataset augmentation

To mitigate class imbalance and enhance the model’s robustness to real-world orchard variability, a targeted data augmentation strategy was implemented. Unlike indiscriminate augmentation, our protocol selectively amplified images characterized by a high floral density. Specifically, augmentation was applied only to images in which the number of apple flower instances was at least twice that of bud instances. This threshold was empirically determined to identify scenarios where the complexity of dense floral clusters necessitated additional training signals.

For each qualified image, five augmented variants were generated to simulate specific environmental perturbations while preserving botanical plausibility:

1. Brightness degradation: Simulating shadows and low-light conditions by reducing the Value channel in HSV space by a factor randomly sampled from [0.5, 0.85].
2. Horizontal flip: Mirroring the image along the vertical axis to account for arbitrary branch orientations.
3. Combined horizontal flip and brightness degradation.
4. Vertical Flip: Mirroring the image along the horizontal axis to reinforce rotational invariance.
5. Combined vertical flip and brightness degradation.

**Figure 7:**
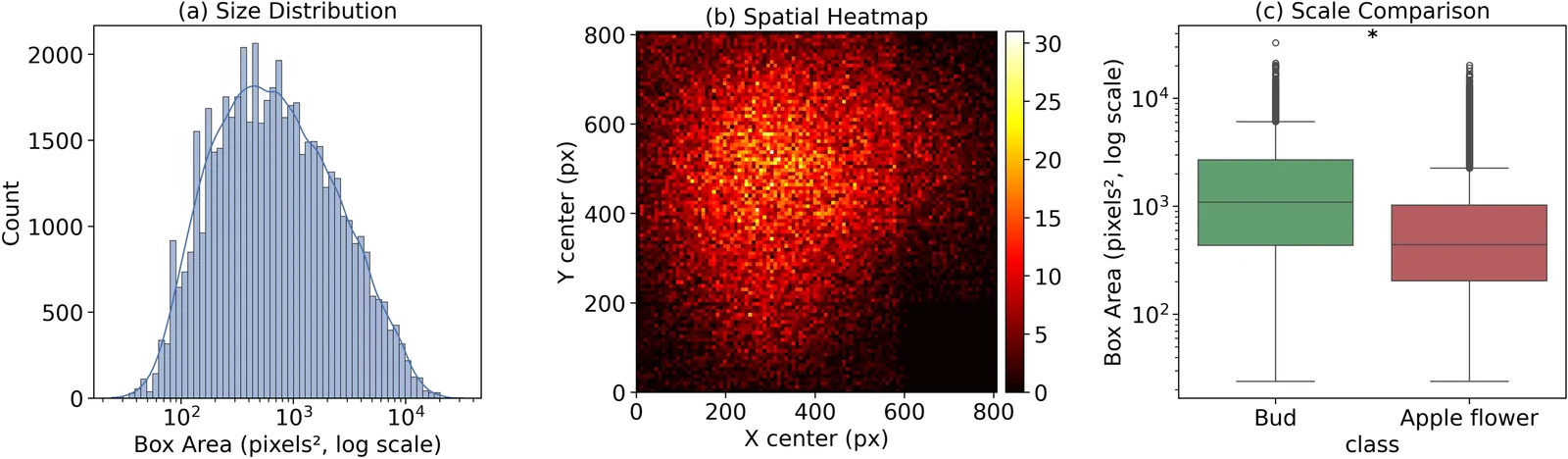
Statistical analysis of the dataset. **(A)** Size distribution histogram of bounding box areas. **(B)** Spatial heatmap indicating the density and distribution of targets. **(C)** Scale comparison box plot between bud and flower classes. The asterisk (*) denotes a statistically significant difference in median area, highlighting the intrinsic scale disparity that informs the model training strategy.

Notably, affine transformations involving rotation or shearing were deliberately excluded to prevent unnatural distortions of the radial symmetry inherent to flowers and buds. Annotations were updated synchronously with the image transformations and retained the original resolution. A representative example of this pipeline is illustrated in **Figure 7**.

Consequently, the dataset expanded to 3,117 images and 52,181 annotated instances. Crucially, the absolute count of apple flowers increased from 13,215 to 23,105, effectively reducing the class imbalance ratio from 2:1 to 1.26:1. The statistical details before and after augmentation are summarized in **Table 2**. This targeted enrichment ensures that the model allocates sufficient capacity to learning the fine-grained features of flowers, which are critical for accurate bloom mapping.

**Table 2:** Statistical comparison of annotated instances before and after data augmentation.

Category	Before augmentation	After augmentation
Apple flower	13,215	23,105
Bud	26,536	29,076
Total	39,751	52,181
Bud-to-flower ratio	2:1	1.26:1

### 3.4 Dataset statistical analysis

**Figure 8:**
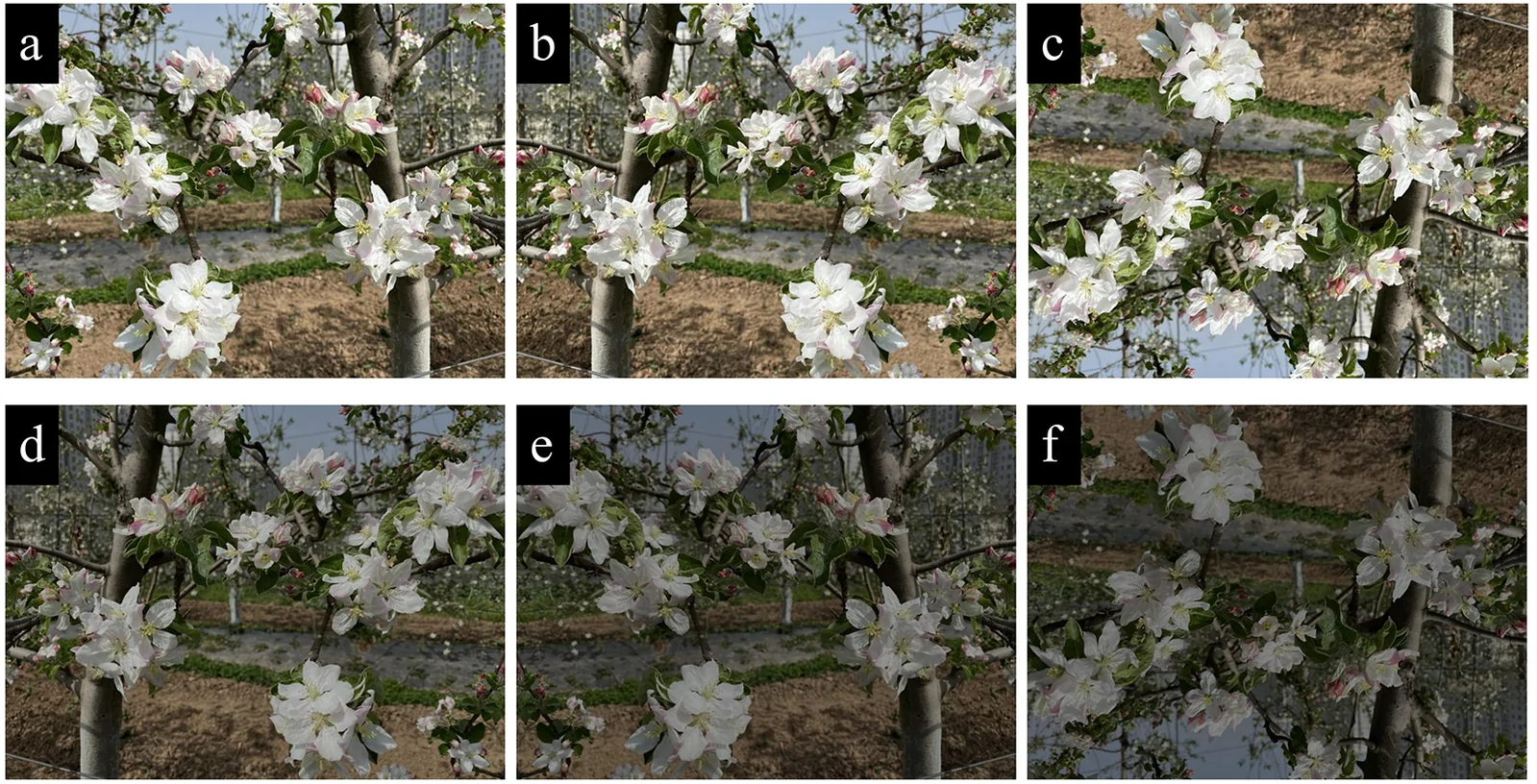
Representative examples of data augmentation. **(A)** Original image; **(B)** Horizontal flip; **(C)** Vertical flip; **(D)** Darkening; **(E)** Horizontal flip + Darkening; **(F)** Vertical flip + Darkening.

To characterize the statistical properties of the curated dataset and inform the subsequent training strategy, a quantitative analysis of bounding box sizes, spatial distribution, and inter-class scale variance was conducted, as illustrated in **Figure 8**.

#### 3.4.1 Size distribution

**Figure 8A** presents the frequency distribution of bounding box areas on a logarithmic scale. The distribution exhibits a unimodal trend peaking between 10^2^ and 10^3^ pixels, with a long tail extending beyond 10^4^ pixels. This indicates that while most targets are small-to-medium sized, the dataset retains a significant number of large instances, posing challenges for multi-scale detection.

#### 3.4.2 Spatial distribution

The spatial heatmap in **Figure 8B** visualizes the center coordinates of all labeled targets. The high-intensity central region signifies a dense clustering of blossoms, which inherently implies severe natural occlusion and overlapping instances in orchard imagery.

Scale comparison between categories: **Figure 8C** provides a box plot comparison of box areas for buds versus flowers. Notably, buds demonstrate a higher median area compared to flowers. This discrepancy is attributed to the annotation protocol: buds are annotated based on the whole visible structure, whereas flowers are anchored to the central stamen. The statistical significance of this difference (marked with *) underscores the intrinsic scale variance between the two classes. This analysis validates the necessity of the targeted data augmentation strategy (Section 3.3) to mitigate class imbalance and ensures the model learns robust features across different scales.

Implications for model training: The aforementioned statistical properties impose specific requirements on the detection architecture and training regimen. First, the predominance of small-to-medium sized targets (**Figure 8A**) necessitates the use of detectors with strong multi-scale feature extraction capabilities. Second, the dense central clustering revealed by the spatial heatmap (**Figure 8B**) implies a high probability of inter-object occlusion, underscoring the importance of the stamen-centric annotation strategy. Crucially, retrospective analysis of these distributions validates the rationale behind the targeted augmentation strategy favoring flower-dominant images (Section 3.3). By quantifying the inherent scale disparity and density patterns, this statistical insight confirms that allocating greater training capacity to the visually complex flower class—as executed in augmentation pipeline—is essential for mitigating class imbalance and enhancing detection robustness.

### 3.5 Dataset validation

To evaluate the quality and generalizability of the proposed dataset, a stratified partitioning strategy was adopted, followed by benchmark experiments using three state-of-the-art object detection frameworks.

#### 3.5.1 Dataset partitioning

**Figure 9:**
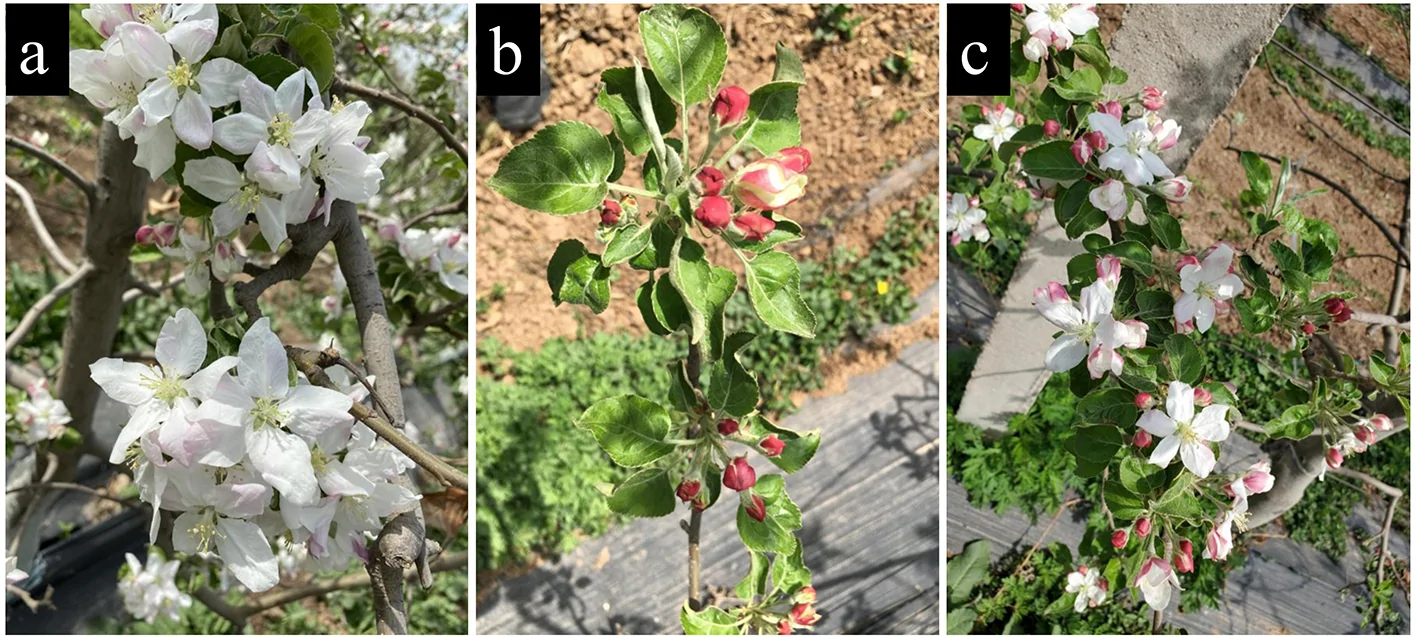
Representative examples of the three compositional categories within the dataset. **(A)** Flower-dominant scenes characterized by fully opened corollas; **(B)** Bud-dominant scenes featuring tightly closed floral buds; and **(C)** Mixed scenes containing both flowers and buds.

To minimize scene-induced bias, the dataset was partitioned using a stratified 8:1:1 split based on the dominant object class per image (i.e., flower-dominant, bud-dominant, or mixed, as illustrated in **Figure 9**). Images were first categorized into these three groups, after which an independent random split was applied within each group. A fixed random seed (seed = 42) was employed to ensure the reproducibility of the dataset partitioning. The detailed statistics of the partitioned dataset are summarized in **Table 3**.

**Table 3:** Detailed statistics of the training, validation, and test sets.

Subset	Images	Instances	Bud	Apple flower	Bud-to-flower ratio
Training	2,493	41,437	23,021	18,416	1.25:1
Validation	310	5,219	2,909	2,310	1.26:1
Test	314	5,855	3,278	2,577	1.27:1
Total	3,117	52,181	29,076	23,105	1.26:1

#### 3.5.2 Experimental platform

The benchmark experiments were executed on a unified computational platform to ensure consistent performance evaluation. The system was equipped with an Intel Core i9-11900H processor, 32 GB of system RAM, and an NVIDIA GeForce RTX 3080 GPU with 16 GB of dedicated video memory. The software stack was deployed on Windows 10, utilizing Python 3.8, PyTorch 2.2.2, and CUDA 11.8.

#### 3.5.3 Benchmarking dataset generalizability

**Figure 10:**
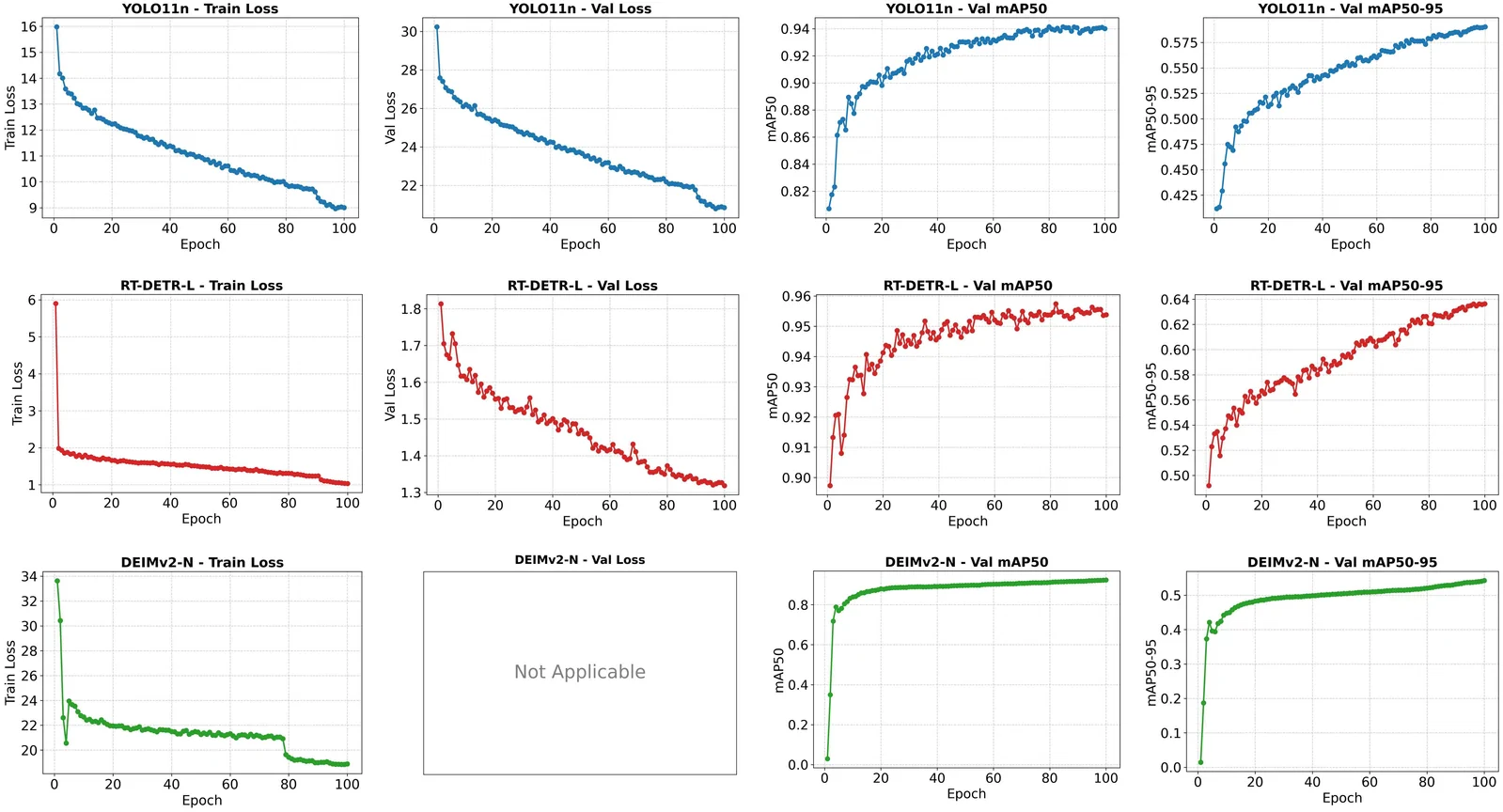
Training and validation dynamics for YOLOv11n, RT-DETR-L, and DEIMv2-N.

To validate the quality and discriminative capacity of the proposed dataset, three architecturally distinct detectors, YOLOv11n (Khanam and Hussain, 2024), RT-DETR-L (Zhao et al., 2024), and DEIMv2-N (Huang et al., 2025), were employed as benchmarking probes. The detailed hyperparameter configurations for these baselines are provided in **Table 4**. To assess the risk of overfitting and validate the dataset’s quality, the training dynamics were comprehensively monitored for all models. **Figure 10** illustrates the corresponding training and validation trajectories, including the convergence of the total training loss as well as the mAP50, and mAP50–95 curves evaluated on the validation set. For YOLOv11n and RT-DETR-L, validation loss was additionally tracked alongside training loss. For DEIMv2-N, while the framework does not compute a conventional validation loss, the validation mAP curves served as the primary generalization indicators. Across all architectures, the training and validation curves exhibit tightly coupled, stable convergence, with validation metrics plateauing or steadily improving in parallel with training performance. This convergent behavior provides robust empirical evidence that overfitting was effectively mitigated under the adopted training configurations. The stable convergence trends observed across all architectures not only validate the effectiveness of the training strategy but also demonstrate the reliability and learnability of the proposed dataset.

**Table 4:** Hyperparameter configurations for the baseline models.

Hyperparameter	epochs	batch size	momentum	Initial learning rate	Weight decay
YOLOv11n	100	8	0.937	0.01	0.0005
RT-DETR-L	100	4	0.937	0.01	0.0005
DEIMv2-N	100	8	–	0.0004	0.0001

To quantitatively assess the detection performance and computational efficiency of the proposed models, four standard metrics were employed: mean Average Precision (mAP), Parameters, Computational cost (GFLOPs), and Model weight size (MB). The primary evaluation metrics are based on Average Precision (AP), which integrates Precision (*P*=*TP*/(*TP*+*FP*)) and Recall (*R*=*TP*/(*TP*+*FN*)) across varying confidence thresholds. Here, *TP*, *FP*, and *FN* denote true positives, false positives, and false negatives, respectively.

The mean Average Precision (mAP) is defined as Equation 1:

(1) $$\text{mAP}=\frac{1}{N}\sum_{i=1}^{N}\text{A}{\text{P}}_{i}$$

where *N* is the number of classes.

mAP50: The mean AP calculated at a single Intersection-over-Union (IoU) threshold of 0.50. This metric emphasizes classification correctness and coarse localization, suitable for rapid screening tasks.

mAP50-95: Following the MS COCO benchmark, this is the average mAP computed over ten IoU thresholds from 0.50 to 0.95 (in steps of 0.05). It serves as a rigorous indicator of localization precision, penalizing even minor deviations in bounding box placement.

Parameters indicate the total number of trainable weights in the model, reflecting storage requirements and model capacity.

Computational cost (GFLOPs) measures the number of Giga Floating Point Operations required for a single forward pass at a given input resolution, characterizing the inference complexity and hardware demands.

Model weight size refers to the disk storage footprint of the trained PyTorch checkpoint (e.g., best.pt), included here as an engineering reference for deployment.

The performance of different models are summarized in **Table 5**. Consistent with expectations, RT-DETR-L achieved the highest performance on this dataset, attaining a mAP50 of 0.957 and a mAP50–95 of 0.648. Its superior performance, particularly on apple flowers (0.970 mAP50), underscores the dataset’s compatibility with state-of-the-art Transformer architectures. However, the notable drop in mAP50–95 relative to mAP50 across all models indicates that precise localization remains challenging, likely due to scale variance and occlusions prevalent in unstructured orchard environments.

**Table 5:** Performance of different detection methods.

Methods	Instances	mAP50	mAP50-95	Parameters/× 10^6^	GFLOPs	Model size/MB	Training time/h
YOLOv11n	All	0.934	0.575	2.6	6.3	15.3	1.26
	Apple flower	0.950	0.587				
	Bud	0.917	0.562				
RT-DETR-L	All	0.957	0.648	32.0	103.4	63.1	4.34
	Apple flower	0.970	0.690				
	Bud	0.943	0.606				
DEIMv2-N	All	0.919	0.540	3.54	6.8	55.1	3.33
	Apple flower	0.939	0.555				
	Bud	0.898	0.526				

YOLOv11n demonstrated remarkable efficiency-accuracy trade-offs, attaining a competitive mAP50 of 0.934 with a minimal model footprint (15.3 MB). This result validates the dataset’s utility for developing lightweight models suitable for edge deployment in precision agriculture. Meanwhile, DEIMv2-N exhibited comparatively lower performance (mAP50: 0.919), suggesting that further architectural refinements are necessary to handle the nuanced features within this dataset.

Crucially, all models consistently performed better on apple flower instances than on bud instances (e.g., RT-DETR-L: 0.970 vs. 0.943; YOLOv11n: 0.950 vs. 0.919). This consistent performance gap serves as an empirical validation of the dataset’s design: buds present an inherently harder detection problem, likely due to morphological similarity to surrounding complex background and higher occlusion rates. Therefore, this dataset not only provides a robust benchmark for current algorithms but also identifies specific pain points for future research.

## 4 Limitations

The primary limitations of this dataset are as follows:

1. A limitation stems from the single acquisition date (March 28, 2021), which captures the orchard exclusively at the early full-bloom stage. This temporal singularity restricts the dataset’s ability to represent the dynamic phenotypic transitions of apple trees, such as the tight-cluster bud break or the progressive petal abscission stages. Consequently, models trained exclusively on this dataset may exhibit diminished accuracy when deployed across varying phenological windows, suggesting a trade-off between high-density annotation quality at a specific stage and broad temporal generalization.
2. Although the use of consumer-grade smartphones enhances accessibility, it introduces uncontrolled optical heterogeneity. Variations in sensor signal processing, white balance, and depth-of-field across the three devices (iPhone 11 Pro Max, 7 Plus, and 6s Plus) create subtle shifts in texture and chromatic features. This heterogeneity increases the difficulty for convolutional neural networks to disentangle object-specific features from device-specific artifacts, potentially explaining the performance gap between models with strong feature extraction backbones (e.g., RT-DETR-L) and lighter architectures (e.g., YOLOv11n).
3. The most profound limitation lies in the biological ambiguity of edge cases. Transitional stages (e.g., buds just commencing anthesis) blur the taxonomic boundary between classes. Despite rigorous expert review, a degree of intrinsic uncertainty remains in these ignore regions or borderline labels. This annotation ambiguity establishes an upper theoretical limit on detection performance.

Future efforts will extend data acquisition across phenological stages and geographic regions, integrate multispectral sensors to mitigate device-induced variability, and employ active learning to refine annotations of ambiguous edge cases, thereby challenging the current performance ceiling.

## Data Availability

The datasets for this study are permanently archived at https://dx.doi.org/10.21227/adt8-4812, and have been mirrored at https://dx.doi.org/10.6084/m9.figshare.33117473 to ensure long-term accessibility.

## References

[B1] Bhattarai U. Bhusal S. Majeed Y. Karkee M. (2020). Automatic blossom detection in apple trees using deep learning. Ifac-Papersonline 53, 15810–15815. doi: 10.1016/j.ifacol.2020.12.216 42574925

[B2] Dias P. A. Tabb A. Medeiros H. (2018). Apple flower detection using deep convolutional networks. Comput. Ind. 99, 17–28. doi: 10.1016/j.compind.2018.03.010 42574925

[B3] Estrada J. S. Vasconez J. P. Fu L. Cheein F. A. (2024). Deep Learning based flower detection and counting in highly populated images: A peach grove case study. J. Agric. Food Res. 15, 100930. doi: 10.1016/j.jafr.2023.100930 42574925

[B4] Farjon G. Krikeb O. Hillel A. B. Alchanatis V. (2020). Detection and counting of flowers on apple trees for better chemical thinning decisions. Precis. Agric. 21, 503–521. doi: 10.1007/s11119-019-09679-1 30311153

[B5] Häni N. Roy P. Isler V. (2020). MinneApple: A benchmark dataset for apple detection and segmentation. IEEE Robot. Autom. Let. 5, 852–858. doi: 10.1109/LRA.2020.2965061

[B6] Huang S. Hou Y. Liu L. Yu X. Shen X. (2025). Real-time object detection meets DINOv3. arXiv preprint, arXiv:2509.20787. doi: 10.48550/arXiv.2509.20787

[B7] Khanam R. Hussain M. (2024). YOLOv11: An overview of the key architectural enhancements. arXiv preprint, arXiv:2410.17725. doi: 10.48550/arXiv.2410.17725

[B8] Li G. Suo R. Zhao G. Gao C. Fu L. Shi F. . (2022). Real-time detection of kiwifruit flower and bud simultaneously in orchard using YOLOv4 for robotic pollination. Comput. Electron. Agric. 193, 106641. doi: 10.1016/j.compag.2021.106641 42574925

[B9] Lu Y. Young S. (2020). A survey of public datasets for computer vision tasks in precision agriculture. Comput. Electron. Agric. 178, 105760. doi: 10.1016/j.compag.2020.105760 42574925

[B10] Mu X. He L. Heinemann P. Schupp J. Karkee M. (2023). Mask R-CNN based apple flower detection and king flower identification for precision pollination. Smart Agric. Technol. 4, 100151. doi: 10.1016/j.atech.2022.100151 42574925

[B11] Sun K. Wang X. Liu S. Liu C. (2021). Apple, peach, and pear flower detection using semantic segmentation network and shape constraint level set. Comput. Electron. Agric. 185, 106150. doi: 10.1016/j.compag.2021.106150 42574925

[B12] Tian Y. Yang G. Wang Z. Li E. Liang Z. (2020). Instance segmentation of apple flowers using the improved mask R–CNN model. Biosyst. Eng. 193, 264–278. doi: 10.1016/j.biosystemseng.2020.03.008 42574925

[B13] Yuan W. (2024). AriAplBud: an aerial multi-growth stage apple flower bud dataset for agricultural object detection benchmarking. Data 9, 36. doi: 10.3390/data9020036 30654563

[B14] Zhao Y. Lv W. Xu S. Wei J. Wang G. Dang Q. . (2024). “ DETRs beat YOLOs on real-time object detection”, in: Proceedings of the IEEE/CVF conference on computer vision and pattern recognition. (CVPR), Seattle, WA, USA: IEEE. 16965–74. doi: 10.1109/CVPR52733.2024.01605

[B15] Zhou W. Cui Y. Huang H. Huang H. Wang C. (2024). A fast and data-efficient deep learning framework for multi-class fruit blossom detection. Comput. Electron. Agric. 217, 108592. doi: 10.1016/j.compag.2023.108592 42574925

